# Approaches to optimize patient and family engagement in hospital planning and improvement: Qualitative interviews

**DOI:** 10.1111/hex.13239

**Published:** 2021-03-24

**Authors:** Natalie N. Anderson, G. Ross Baker, Lesley Moody, Kerseri Scane, Robin Urquhart, Walter P. Wodchis, Anna R. Gagliardi

**Affiliations:** ^1^ Toronto General Hospital Research Institute University Health Network Toronto Canada; ^2^ Institute of Health Policy, Management and Evaluation University of Toronto Toronto Canada; ^3^ Princess Margaret Cancer Centre University Health Network Toronto Canada; ^4^ Patient Partnerships University Health Network Toronto Canada; ^5^ Department of Community Health and Epidemiology Dalhousie University Halifax Nova Scotia Canada

**Keywords:** hospital planning, hospitals, patient engagement, patient participation, patient‐centred care, qualitative research, quality improvement

## Abstract

**Background:**

Patient engagement (PE) in health‐care planning and improvement is a growing practice. We lack evidence‐based guidance for PE, particularly in hospital settings. This study explored how to optimize PE in hospitals.

**Methods:**

This study was based on qualitative interviews with individuals in various roles at hospitals with high PE capacity. We asked how patients were engaged, rationale for approaches chosen and solutions for key challenges. We identified themes using content analysis.

**Results:**

Participants included 40 patient/family advisors, PE managers, clinicians and executives from 9 hospitals (2 < 100 beds, 4 100 + beds, 3 teaching). Hospitals most frequently employed collaboration (standing committees, project teams), followed by blended approaches (collaboration + consultation), and then consultation (surveys, interviews). Those using collaboration emphasized integrating perspectives into decisions; those using consultation emphasized capturing diverse perspectives. Strategies to support engagement included engaging diverse patients, prioritizing what benefits many, matching patients to projects, training patients and health‐care workers, involving a critical volume of patients, requiring at least one patient for quorum, asking involved patients to review outputs, linking PE with the Board of Directors and championing PE by managers, staff and committee/team chairs.

**Conclusion:**

This research generated insight on concrete approaches and strategies that hospitals can use to optimize PE for planning and improvement. On‐going research is needed to understand how to recruit diverse patients and best balance blended consultation/collaboration approaches.

**Patient or public contribution:**

Three patient research partners with hospital PE experience informed study objectives and interview questions.

## 1 BACKGROUND

Patient (and family) engagement (PE) is defined as patients, families or their representatives, and health professionals working in active partnership at various levels across the health‐care system—individual care, organizational design and governance, and system‐level policy‐making—to improve health and health care.^1^ PE in health‐care organization planning and improvement (henceforth, hospital PE) is a growing practice that can lead to a range of beneficial impacts such as educational tools for patients, programmes and services tailored to patient needs and preferences, enhanced patient experiences and better clinical outcomes such as reduced admissions.^2^, ^3^ However, many barriers can result in token PE, and little or no service improvement. For example, a systematic review (26 studies 2000‐2015) found that key barriers resulting in token PE included uncertainty among patients about their role and resistance from clinicians to working with patients.^4^ Another systematic review (11 studies 2003‐2012) found that patients were typically consulted after decisions had been made, which did not lead to improvements.^5^ More recently, a systematic review of 42 reviews (up to 2018) identified numerous organizational barriers of PE: knowledge, attitudes, expectations, communication, financing, resourcing, training, patient/family recruitment and representation, and addressing power dynamics.^6^

By synthesizing research to date, these reviews identified gaps in knowledge about how to optimize PE in health‐care organization planning and improvement including strategies to capture diverse perspectives and approaches to engage patient (and family) advisors. It is not clear whether more active engagement approaches involving collaboration (patients and providers working together to create solutions) are necessary for all planning and improvement decisions and whether collaboration always leads to improved programmes or services, patient experiences or clinical outcomes compared with less active engagement approaches such as consultation (patient opinions or ideas sought via survey, interview or focus group).^2^ It has been proposed that employing a ‘mosaic’ of engagement approaches is best because it alleviates the expectation that a few select patients can represent the voices of all patients, and that including many voices through different types of engagement allows for a more robust understanding of patient needs and preferences.^7^, ^8^

While PE is needed in all sectors, data on approaches in the hospital sector remain limited.^4^, ^5^, ^6^ Hospitals provide inpatient, outpatient and emergency services, and account for the largest share of health spending in many countries.^9^ In a scoping review, we included only 10 studies published in 2016 or earlier that focused on PE in hospital planning and improvement.^3^ Included studies provided little detail about precisely how patients were engaged. For example, a survey of hospital quality managers found that 50% of hospitals engaged patients, and in 65% of those hospitals, patients were members of quality committees, but the survey did not gather specific information such as mode or frequency of patient engagement, what information they contributed, and how it was used and with what impact.^10^ Given little evidence‐based guidance on how to best translate the patient voice in improving hospital services, experiences and outcomes, the purpose of this study was to generate insight for optimizing PE in hospital planning and improvement. The objective was to explore approaches and strategies used to engage patients in hospitals recognized for PE capacity including infrastructure and activities. Those best practices could be used in future by hospitals to develop their capacity for PE in organizational planning and improvement decisions.

## 2 METHODS

### 2.1 Approach

We chose a qualitative research design to thoroughly explore PE practices and conducted qualitative interviews with individuals involved in PE at hospitals with high PE activity.^11^ We employed a qualitative descriptive approach, which does not test or generate theory, but instead explores views and experiences to identify barriers to, and suggested solutions for improving health services.^12^ We complied with standards for reporting qualitative research and enhancing rigour.^13^, ^14^ We acquired ethics approval through the University Health Network Research Ethics Board. The research team, including four health services researchers, three patient research partners with PE experience at different Ontario hospitals, two patient engagement managers, a biostatistician and representatives of the Ontario Ministry of Health, Ontario Hospital Association and Canadian health‐care accreditation agency, contributed to research design and planning, question development, data analysis and interpretation of the findings. All participants provided written informed consent prior to interviews. There was no prior relationship between the researchers and participants.

### 2.2 Sampling and recruitment

We used purposive sampling to recruit individuals whose PE views and experiences might vary by role (managers responsible for PE, or patients/family or clinicians involved in at least one PE project), type of hospital (<100 beds, 100 + beds, teaching) and health‐care region in Ontario, Canada. We also used snowball sampling by first interviewing PE managers, who referred us to patients/family and clinicians. We recruited participants from hospitals with high PE capacity, identified by a survey of hospital PE managers that we had administered in the year prior to these interviews.^15^ High PE hospitals were those that featured PE in planning and improvement activities across multiple clinical and corporate departments and employed a variety of engagement approaches. We aimed to recruit 1 PE manager, 2 patient/family and 2 clinicians from 2 hospitals of each type for a minimum total of 30 interviews. We first contacted PE managers by email on 13 January 2020 and closed recruitment on 16 July 2020. Sampling was concurrent with data collection and analysis, and ceased when, through discussion, the research team agreed that thematic saturation was achieved.

### 2.3 Data collection

We conducted interviews by telephone between 21 January and 16 July 2020. NA (MPH, Research Associate) and ARG (PhD, Senior Scientist/Professor) jointly conducted the first two interviews, independently reviewed transcripts and then met to discuss and refine wording of interview questions. NA subsequently conducted all interviews. Interview guide questions (Data S1) aimed to elicit the rationale for, and barriers of, PE approaches, distinguished according to prior research as involving more intensive (collaboration: joint decision making on project teams or standing committees) versus less intensive engagement (consultation: gathering ideas or feedback using surveys, interviews or focus groups).^2^ Questions were reviewed and refined by the research team prior to use. We first asked participants to describe a hospital planning or improvement activity that engaged patients in some way that they were involved in or aware of (reported elsewhere), and refer to that as a reference for subsequent questions: How were patients engaged, why was that PE approach chosen, what strategies were used to ensure patient input was used, what were key barriers and solutions, and what would you do different in future to optimize PE? Interviews ranging from 21:38 to 73:29 minutes were audio‐recorded and transcribed.

### 2.4 Data analysis

We employed content analysis to identify themes inductively through constant comparison and used Microsoft Office (Word, Excel) to manage data.^11^ NA and ARG independently coded the first two interviews and then discussed coding to develop a preliminary codebook of themes and exemplar quotes (first level coding). NA coded subsequent interviews to expand or merge themes (second level coding). NA and ARG met on two subsequent occasions to review, discuss and refine coding. We tabulated data (themes, quotes) by participant role and hospital type to compare themes. The research team reviewed themes and quotes. We used summary statistics to describe participants and text to describe key themes.

## 3 RESULTS

### 3.1 Participants

We interviewed 40 participants (Table 1). By role, they included 20 patient/family advisors, 10 clinicians, 8 PE managers and 2 corporate executives. Participants were affiliated with 9 hospitals including 2 < 100 beds (8 participants), 4 100 + beds (21 participants) and 3 teaching (11 participants). Five hospitals (2 < 100 beds, 2 100 + beds, 1 teaching) reported 3 to 5 years of PE experience, and 4 hospitals (2 100 + beds, 2 teaching) reported 6 or more years of PE experience. Patient/family advisors had a mean age of 66.2 years, 75.0% were women, and 90.0% identified as Caucasian. Clinicians were 90.0% women and all were mid‐ or late career. Clinician specialty included 1 physician, 6 nurses, 1 social worker and 2 occupational therapists. PE managers had a mean of 10.9 years of experience in PE roles, and 75.0% were women. One corporate executive was a woman, and one was early career, and the other was mid‐career.

**TABLE 1:** Participant characteristics

Role	Affiliation by hospital type			Sub‐total
	<100 beds	100 + beds	Teaching	
PE managers	2	4	2	8
Patient/family advisors	4	10	6	20
Clinicians	2	6	2	10
Corporate executives	0	1	1	2
Sub‐total	8	21	11	40

Data S2 includes data, and themes with selected quotes are discussed here. Notably, there were no clear discrepancies in themes articulated by role.

### 3.2 PE was embedded throughout organizations

Participants said that patients were involved in decision making for all hospital activities.

We always have a patient or two involved in everything that we do (038 exec teaching)

We sit on all committees in the hospital (002 patient/family <100)

PE was considered important because it allowed health‐care workers to see issues with a patient lens, resulting in better understanding of patient needs and preferences, and services reflecting those perspectives. This was believed to lead to improved patient experiences and outcomes. PE was therefore described as essential to patient‐centred care.

They [healthcare workers] see things through new eyes (036 patient/family <100)

Being able to see our system through their eyes is very informative and leads to better patient experiences which also leads to better outcomes (025 clinician 100+)

To have patient‐family centred care, you can’t just pay lip service to it, you absolutely have to engage patients (039 patient/family teaching)

### 3.3 Engagement structures

Participants said they engaged patients through a variety of structures including patient and family advisory councils (PFACs), standing committees and project teams.

#### 3.3.1 General and specific PFACs

All hospitals had a general PFAC, and 100 + bed and teaching hospitals also had PFACs for specific clinical units such as the neonatal intensive care unit (NICU) and mental health department.

I lead our Patient and Family Advisory Council within our Mental Health Department (034 clinician 100+)

And we partnered with our Parent Advisory Council within NICU (022 clinician 100+)

#### 3.3.2 Standing committees

Participants mentioned numerous committees at the clinical unit/department and corporate levels featuring at least one patient advisor. Membership was continuous and on‐going, meaning the patient advisor contributed to multiple planning or improvement activities over time.

There is a patient on the General Medicine Quality Committee and the Quality Committee…both of those committees had input into what was on the Board agenda (012 clinician teaching)

We have patient and family advisors embedded in…corporate committees but also at the program level committees (032 exec 100+)

#### 3.3.3 Project teams

Participants also said that patients were included in project teams with finite timelines formed to address specific initiatives at both the clinical unit/department and corporate levels. These were sometimes referred to as working groups or steering committees.

We had a small working group that consisted of about 6‐people including a patient and family advisor that helped drive and steer the organization in terms of developing the strategic plan (004 PE manager teaching)

The goal of the stroke council working group was to implement a stroke unit. We had monthly meetings that I was involved in and my role is to bring the patient perspective to these meetings (039 patient/family teaching)

### 3.4 Engagement approaches

Participants largely employed either collaboration, consultation or a blended approach to engage patients, and provided rationales for and examples of those approaches (Table 2). A few participants said that the approach chosen for a PE project would depend on the situation or nature of the project including the issue the project was focusing on, the willingness and commitment from those who would be involved and project time frame. Regardless of which engagement approach was used, participants agreed that in‐person interaction was preferred because it established rapport between patients and health‐care workers, enabled staff to see patients as real people and nurtured an appreciation for the importance of involving patients in planning and improvement.

**TABLE 2:** Comparison of engagement approaches

Themes	Engagement approach		
	Consult	Collaboration	Blended approach
Use	Common	Most common	Less common
Purpose	Gather feedback about new or existing programmes, priorities for strategic planning, or ideas to plan or improve services	Discuss or evaluate issues, brainstorm or develop solutions, and create, review or edit documents and resources	Use co‐design to validate and elaborate on ideas identified in consultation
Methods	Surveys, interviews, world cafés, focus groups, post‐discharge phone calls	Regular in‐person or virtual project team and standing committee meetings	See consult and co‐design
Rationale	Reaches many patients Captures diverse perspectives Efficient way to rapidly gather information in support of decision making	Ensures patient perspectives heard and integrated in decision making Evidence‐based/credible Mandated by management	Consultation gathers a wide range of perspectives, and then, co‐design provides deeper insight on those ideas and which might be prioritized because they would lead to the biggest improvements
Examples	A group of about 200 patients and families who have had care in the organization have agreed to be a part of a virtual group where they would receive a few surveys a month on various topics (028 PE manager teaching) They [the hospital] had several booths set up for two weeks in the main corridor. They invited patients or whoever was in the main lobby. So getting their feedback to enable creating this new strategic plan (005 patient/family teaching) We were given a certain number of questions and we were calling people just to get feedback. They [patients] would give their feedback on the phone. We [Patient Experience Partners] would enter that information and then that would go to the coordinators of the project and it would also go to the managers of the unit. And that was all about trying to improve the quality of care (018 patient/family 100+)	I sit on a really new innovative programme. It was brand new way of delivering services at the hospital, and myself and the other patient partner would for sure say that we influenced the way that that programme was developed (014 patient/family teaching) Myself and about five or six other patient advisors were involved in the development of an online tool for seniors regarding their health. We met regularly to develop the content that would be included on each page of this site down to the visuals, the sounds of the voice prompts and anything that we felt was relevant (019 patient/family 100+) So in the NICU, we took an opportunity to revamp a parent information booklet. We actually had parents help us define what the key elements of the information brochure and/or platforms would be in terms of paper‐based and electronic version. They helped us to develop the table of contents and what the important pieces were that needed to be included (022 clinician 100+)	We involved patients and the families in what they wanted to put on whiteboards and what they would look like. We did informal surveys with patients in the hospital. We brought different samples of the whiteboards to our PFAC for them to have input (001 PE manager <100) The overall results [of patient surveys about daily rounding] came back to the patient and family experience steering committee to inform whether we needed to make some tweaks to the process that we would then trial in our next PDFA cycle (024 PE manager 100+)

#### 3.4.1 Collaboration

Most projects described by participants involved collaboration approaches. Most commonly, the purpose of collaboration was to partner or collaborate with patients in creating, reviewing or editing documents or resources such as patient information handouts or videos, online educational tools, web sites or procedure consent forms. Collaboration was also employed to discuss and evaluate issues pertaining to planning or improving services or programmes, brainstorm or develop solutions for those issues, and to inform the development of innovative new programmes. Collaboration methods included monthly or bimonthly project team or standing committee meetings held virtually or in‐person. Participants who preferred collaboration offered three reasons. Some said it was the best approach for ensuring that patient perspectives were heard and integrated in decision making.

Having people at the table through all of the discussions was extremely important with respect to making sure the voices of patients were there at all times (028 PE manager teaching)

Participants said they used collaboration because it was evidence‐based, referring to their own past experience or successful use in other hospitals.

Co‐design is actually an established practice…co‐design has been shown to be very successful (014 patient/family teaching)

We did ask what some of the other hospitals are doing, the [patient and family advisory committees] at other hospitals. They had experiences and approaches…we did consider a few and decided on the approach that I mentioned (032 exec 100+)

Participants also said that collaboration was expected or mandated by PE managers, hospital leaders or the Board of Directors.

It’s within our corporate goal. It’s mandatory that they’re partnering with patients and families and they have to be able to demonstrate that, through partnership, they have been successful (010 PE manager 100+)

Our hospital, well they almost always mandate that patient and family advisors have to be on these kinds of committees (003 patient/family teaching)

#### 3.4.2 Blended approaches

Many participants described using blended approaches, though less frequently than collaboration. Blended approaches involved both collaboration and consultation for the same initiative. A blended approach was used to develop patient tools (eg communication whiteboards) and new programmes or models of care (eg daily rounding, post‐discharge contact). In a few instances, collaboration was first used to develop a project, and then, consultation was used as a form of pilot test to gather feedback from other patients or family. More commonly, collaboration approaches followed consultation approaches. In this scenario, the purpose of a blended approach was to use collaboration as a means of validating and elaborating on ideas identified during consultation.

You can’t collaborate with the whole community but you can get their input and then bring it into an environment where collaboration is possible (004 PE manager teaching)

The rationale provided is that consultation through methods such as surveys gathered a wide range of perspectives on a topic, and then, collaboration with patients on project teams or standing committees provided deeper insight on those ideas, which should be prioritized because they would lead to the biggest improvements, and how to design or implement them.

We [patient/family advisors] would break into groups and review the suggestions from the survey group to make sure that it was captured (015 patient/family teaching)

#### 3.4.3 Consultation

Consultation approaches were less frequently used than collaboration or blended approaches. The purpose of consultation was to gather feedback on existing or newly implemented programmes, or ideas about how to plan or improve services. Consultation methods included surveys, interviews, focus groups and post‐discharge telephone calls. Participants who preferred consultation offered three reasons. Some said consultation was the best approach for reaching many patients.

We attended five or six summer festivals and choosing that style allows us to get many perspectives instead of just one or two. We had thousands and thousands of points of data (004 PE manager teaching)

The broader the input, the better it is…it’s getting as much information from as many people as possible (030 patient/family 100+).

Participants said that a single person cannot represent the myriad of perspectives captured through consultation with patients with varied characteristics or experiences.

A few [patient/family advisors] at the table is a heavy burden for those individuals to carry the voice of all patients (028 PE manager teaching)

Participants also valued consultation because it was an efficient way to rapidly gather feedback for making improvements or solving problems as they arise.

They [staff] want to get information from patients faster so that they can look at making decisions about improvements more immediately (018 patient/family 100+)

### 3.5 Strategies to optimize engagement

Participants described numerous strategies employed to ensure that multiple and diverse perspectives were sought, heard and integrated in decision making (Table 3).

**TABLE 3:** Strategies used to optimize engagement

Theme	Exemplar quotes
Engage diverse patients	Aim for diversity in characteristics We believe in making sure that the most marginalized individuals have represented voices at the table…one of the things in terms of how we've been successful with being able to recruit these types of individuals is that we have a strategy around recruiting for diversity. So we specifically are looking for folks that represent the health disparities in our community and engage them (010 PE manager 100+) Employ various recruitment strategies to achieve diversity A lot of our recruiting often times is by word of mouth and we've tried newspaper ads, the last one we got through Facebook…you know it's those kinds of things; how do you reach the biggest population? (036 patient/family <100) Patient/family advisors were largely retired persons It tends to be the retired community that comes forward to be part of the patient and family advisory committee (027 PE manager <100) Because I’m retired I’m able to give the time to things (023 patient/family 100+)
Prioritize what benefits many	Chose projects that benefit the majority A lot of suggestions come to PFAC, but if they are more individualized, we try to triage that because it's not about one, it's about everybody. We try to talk about who is our catchment and who are we benefiting (002 patient/family <100) Used perspectives expressed by the majority And then when the structured reviews came back from patients and their families we had to set a priority, we're going to take everything that is said by more than you know ‘x’ percentage of patients and we're going to use that (012 clinician teaching) Blended approach of consultation then co‐design We were really trying to be driven by the data from our survey and post‐discharge phone calls, and then validating that with the experiences of our patient and family partners to dig a little bit deeper on some things that would have the biggest impact on improving our results (024 PE manager 100+)
Match patients to projects	Deploy those with PE experience/skill Sometimes we were selected because of other projects we had worked on. I mean they [PE managers/staff] have a good sense of our skills at this point. There's a large number of patient partners but there seems to be a group that does a lot of different kinds of projects and so they know who's got good analytical skills and good communication skills. So we were sought. We were recruited specifically (014 patient/family teaching) Match patient/ family experience or characteristics to PE project And basically what they ask for generally is people who have had a background as a patient in those areas. So for instance, when things come out in the neuro area or the cancer area I wouldn't apply because my background there as a patient just doesn't exist (039 patient/family teaching)
Train participants	Train patient/family for role of advisors They are trained during orientation and then have, with the interest in the program, a full day of training and then continued engagement throughout the program (037 clinician < 100) We usually have an education session for about a half an hour before we get into the meeting (036 patient/family < 100) Train health‐care workers on how to collaborate with patient/family advisors The staff and leaders received training on how to effectively engage with patient partners (029 patient/family 100+)
Ensure patient perspectives inform decisions	Include a critical volume of patient/family advisors About four years ago we established our first patient and family experience steering committee. The initial membership, the staff greatly out‐numbered the number of patient family partners. Over the last few years we've decreased the number of staff on the committee, increased the number of patient family partners on the committee (024 PE manager 100+) Quorum requires at least one patient/family advisor There's usually two of us [patient/family advisors on standing committees]. One of the requirements of the [Research Ethics Board] is that to have a proper quorum you need to have one patient/family advisor at the meeting (17 pat 100+) PFAC review of standing committee or project team work I would report back to the Patient and Family Council about what is going on in the General Medicine Quality Committee…and sometimes I’d be seeking out advice (007 patient/family teaching) Patient/family advisor feedback loop When we [patient/family advisors] made those suggestions, they were taken away and then at the next meeting they would hand the draft out and we'd go over it to see which of our suggestions had been included (035 patient/family <100) Somebody comes back to you and says, here's how your comments changed what we did. It's a very simple feedback loop but it's made a big difference (014 patient/family teaching) We're sending that out so that they [patient/family advisors] look at it and make sure that it reflects what they've said (040 clinician 100+) Philosophical commitment to respect/value patient perspective We've evolved and developed an understanding that any member around the table would have equal input or equal weighting to their opinions. So whether or not it was a frontline staff at the table or a patient and family advisor or the vice‐president or a physician (004 PE manager teaching) We come to the table with the philosophy that our family advisors are the experts (008 clinician teaching) Everybody had an equal say at that table and their comments were very well received so nobody was hesitant to speak up (023 patient/family 100+) The respect for patient/family advisors within the whole organization is very conducive to them [healthcare workers] listening to us and taking our advice (003 patient/family teaching) We've come to value that. They are like team members. It's built into the organization (020 PE manager 100+)
Staff champions	Skilled PE managers/staff They have really key skills around hearing, deep listening and reflective listening skills, and know the importance of being able to hear and integrate (028 PE manager teaching) Proactive standing committee/project team Chairs The chair of the working group made sure that all members are actively participating and that their voice is heard (038 corporate executive teaching)
Links with Board of Directors	Board member on PFAC Having a Board member sit on the PFAC, and bring those minutes to the Board [Quality] Committee and to the full Board ensures that if they need different equipment or whatever, that it's not just being minuted in a meeting and then never done (001 PE manager <100) Patients on Board/Board Committees We [patient/family advisors] also have voting rights on the Board committees (036 patient/family <100) Accountable to Board Being accountable to the Board…to report that there has been implementation and change (037 clinician <100)

#### 3.5.1 Engage diverse patients

Participants said that their hospitals aimed to recruit diverse patients who varied by role (ie patient, family member) and other characteristics of the community they served, emphasizing diversity and those with health disparities. Patients were recruited in various ways including social media, email, newspaper ads, word of mouth, call‐outs and posting formal job descriptions. Despite the emphasis on seeking diversity, most members of patient/family advisory committees were retired persons who had time for PE activities, potentially limiting the extent to which planning or improvement decisions were informed by diverse perspectives.

#### 3.5.2 Prioritize what benefits many

Participants said that they reviewed a wide range of patient feedback, but prioritized ideas for planning and improvement based on what was likely to benefit the majority of individuals in the community they served. Complementary to this was the strategy of first using consultation approaches to capture a wide range of ideas from many patients, followed by collaboration approaches involving select patients to prioritize and elaborate on ideas. Both of these approaches may not capture the perspectives of underserved or marginalized community members.

#### 3.5.3 Match patients to projects

Participants described various ways of allocating patients to projects, but differed in how this was defined. Some participants said it was important to match patient experiences or characteristics to a project, while others said they deployed PFAC members with PE experience and skills to multiple projects, a strategy that might limit diversity.

#### 3.5.4 Train participants

Once recruited, patients were prepared for PE roles through general orientation and then further education in advance of assuming membership on committees or project teams to provide patients with background on committee or project activities. Some participants also said that health‐care workers received training on how to effectively engage with patients.

#### 3.5.5 Ensure patient perspectives inform decisions

The most commonly mentioned factor supporting PE was organization‐wide respect for patient perspectives that had developed over time such that PE was the accepted norm. Participants said their hospitals had developed a philosophical commitment that patient/family advisors are experts on the patient perspective and their perspectives were valued equally to those of health‐care workers. Given that 5 hospitals had 3 to 5 years of PE experience, and 4 had 6 plus years, perhaps organizational commitment to PE may be just as or perhaps more important than length of time.

Participants described several additional strategies for ensuring that patient perspectives were heard and informed decisions. One strategy was to include a critical volume of patients on committees or project teams so that they were not outnumbered by health‐care workers. Another strategy was to require at least one patient in quorum, or the minimum number of individuals who can form decisions. An alternate strategy was for the PFAC to review interim progress, decisions or outputs of standing committees or project teams to provide advice or support, and further ensure that patient perspectives were integrated in planning and improvement. Yet, another strategy was to follow up with patients to confirm that interim or near‐to‐final decisions or outputs accurately captured their perspectives, and upon review, patients could offer further feedback. Such feedback loops were valued by patient/family advisors who could see how their perspectives contributed to planning and improvement.

#### 3.5.6 PE manager/staff champions

Health‐care workers at various levels promoted and supported the use of patient perspectives in decisions. PE managers championed the involvement of patient/family advisors in hospital activities. PE staff possessed the abilities to listen, tease out patient perspectives and integrate them. Chairs of standing committees or project teams proactively consulted with patient/family advisors throughout meetings to ensure they understood what was being discussed, ask if they had any questions, or wanted to articulate ideas or feedback.

#### 3.5.7 Links with Board of Directors

Linking PE activity with the Board of Directors was another important strategy for ensuring patient perspectives informed decisions. This was achieved in several ways including a Board member on the PFAC so that they could convey concerns or ideas directly to the Board, including patients on the Board or Committees of the Board as voting members and making the PFAC accountable to the Board for planning and improvement activities.

### 3.6 Engagement challenges and recommended solutions

When asked what they would do differently in the future to further optimize engagement, participants articulated several recommendations. One recommendation pertained to recruiting a larger pool of patients given the ever‐increasing number of planning or improvement projects.

We’ve had to increase the number of advisors because we’re being asked to be involved in many different projects and we just don’t have enough people (005 patient/family teaching)

Consider gathering a larger group of patient and family advisors so we have a bigger pool of resources (034 clinician 100+)

Participants also underscored the need to recruit patients with a range of characteristics and experiences, though not specifically how. This would achieve two stated strategies for optimizing engagement: ensuring a diversity of perspectives that represent the community and matching patients to projects based on characteristics and/or experiences. Participants noted this was particularly challenging, resulting in the redundant deployment of the same few patients whose perspectives reflected retired persons on multiple committees/teams.

I’m a semi‐retired white male and I don’t really feel I represent my community at the hospital. There needs to be more effort made to bring people to the table that aren’t necessarily system savvy and engagement literate (029 patient/family 100+)

Some participants recommended engaging patients earlier in planning or improvement activities via collaboration instead of asking patients to comment on decisions already made by health‐care workers so that decisions would better reflect patient perspectives, and to save time by avoiding multi‐step, iterative processes.

We’ll have already done all the work and we’ll bring it to a PFAC group for review and we could have saved so much time if they’d been involved at the very beginning (038 exec teaching)

The people in this particular clinic have decided they’re going to do ‘x’ and ‘y’. I thought the new decisions were not great. I wish there was more opportunity for co‐design because that could have saved some problems that we were trying to solve later (014 patient/family teaching)

## 4 DISCUSSION

This study found that hospitals selected for high PE capacity had embedded PE activities broadly, engaging patients throughout the organization in many planning and improvement decisions via multiple structures including standing committees, project teams, and general and unit/department‐specific PFACs. Participants most frequently employed collaboration approaches (membership on standing committees/short‐term project teams), followed by blended approaches (both collaboration and consultation), and, less frequently, consultation (surveys, interviews). They described a wide range of strategies that supported engagement approaches to ensure that diverse perspectives were sought, heard and integrated in planning and improvement decisions, but faced challenges in achieving this goal.

Prior research that referred to PE largely focused on engaging individuals in their own clinical care or as members of research teams.^16^, ^17^ Other research on PE in health‐care organizations was conducted in the primary care context, revealing numerous barriers.^4^, ^5^, ^6^ Little prior research examined organizational PE in hospitals. Malloggi et al surveyed 213 health‐care workers in a French university hospital, revealing they had engaged patients in developing care pathways, patient education programmes and continuing education of health‐care professionals.^18^ A few studies, like ours, were based on qualitative research designs and identified numerous barriers of PE in quality improvement activities in various settings.^19^, ^20^, ^21^ A scoping review of PE in hospital planning and improvement included only 10 studies, which largely described barriers of engagement.^3^ Thus, our research is unique from prior research in that it focused on PE for planning and improvement specifically in hospital settings, and rather than focusing only on barriers, builds on prior research by providing more thorough insight on concrete approaches and strategies to optimize PE that can be broadly applied by other hospitals.

With respect to engagement approaches, most of our participants employed either collaboration or blended approaches. Some who employed collaboration said that it was more likely to ensure that patient perspectives informed decisions, but others said they used collaboration because it was thought to be evidence‐based and therefore mandated by their organization. Those who largely employed blended approaches emphasized they could gather a wide range of ideas or perspectives through surveys or interviews, and then collaborate with patients on project teams or standing committees to prioritize, and then design or implement those ideas. Research to date shows that organizational PE generates practical tools (eg patient handouts) and tailors services to patient needs and preferences, but evidence on the link between PE and improved patient experiences or clinical outcomes is limited.^2^, ^3^ Therefore, we lack knowledge on whether collaboration, consultation or a blended approach is superior. Rather than a ‘mosaic’ of approaches, thought by some to be ideal,^7^, ^8^ blended approaches were less frequently employed than collaboration, and typically involved consultation to first gather a range of perspectives, and then collaboration to prioritize and elaborate on ideas or feedback. Intuitively, the blended approach makes sense, but is the most time‐ and work‐intensive. Furthermore, prioritizing issues that are relevant to the majority by a small group of patient/family advisors may not result in services or programme that reflect the needs and preferences of marginalized community members. Thus, further research is needed to establish which approach is best suited to different types of projects, decisions or desired impacts or outcomes.

With respect to strategies that support engagement, this study revealed numerous ways to ensure that planning and improvement incorporate diverse perspectives. Some of those map onto Oostendorp's Measuring Organizational Readiness for Patient Engagement (MORE) framework, including using PE throughout the organization, training health‐care workers in PE, recruiting patient representatives and preparing patients for their PE role.^22^ This research identified many other ways to support engagement that could be used to elaborate MORE, and applied by hospitals to enhance their PE capacity: engage diverse patients, prioritize what benefits many, match patients to projects, involve a critical volume of patients on committees/teams, require at least one patient to establish quorum, ask involved patients and PFAC members to review outputs, coach PE managers/staff and committee/team Chairs to champion PE and link PE activities with the Board of Directors. Moreover, hospitals with 3 to 5 years of PE experience exhibited a similarly broad range of PE activities and supports compared to hospitals with 6 or more years of PE experience, suggesting that organizational enthusiasm and capacity for PE may be more critical than years of experience. Further research might test or evaluate which strategies and supports optimize engagement, thereby providing hospitals with even further insight on PE capacity.

In our study, patient/family advisors were largely retired Caucasian women, who were deployed to multiple projects. If widespread, then collaboration or blended approaches could lead to unintended consequences, including widening disparities in health and health care. In other words, if the patient/family voice is relatively homogenous (or lacks diversity), and we focus on what benefits the majority of our constituents, then we risk increasing the inequities that currently exist for underserved and marginalized populations. Our participants said there was a need to recruit a larger, more diverse group of patient/family advisors, but did not elaborate on specific strategies for doing so. Some guidance is available from research on involving persons from diverse and hard‐to‐reach communities in research.^23^ Recommendations for recruitment included use of existing networks, consulting with the community, accessing outpatient clinics and using social media; and for supporting engagement included using culturally appropriate communication, building rapport between members, equalizing member roles, establishing trust as the basis for long‐lasting partnerships and establishing a diverse leadership team. On‐going research is needed to identify the infrastructure and processes hospitals must implement to have the capacity to recruit, prepare and support a large, diverse group of patient/family advisors, or, alternatively, how to efficiently and rapidly do so as the need arises.

Strengths of this research included the use of robust qualitative methods that complied with reporting criteria and standard techniques for ensuring rigour.^11^, ^12^, ^13^, ^14^ The research was guided by multiple points of input and review by an interdisciplinary research team that included two patient research partners with hospital PE experience. Furthermore, we interviewed persons with considerable PE expertise and experience from hospitals with proven PE capacity. Participants represented different roles (patient/family, PE managers, clinicians) and hospital types. We must also acknowledge some limitations. Patient/family advisors were not diverse, consisting largely of retired Caucasian women, and the clinicians we interviewed included only one physician. Patient/family advisors were largely PFAC members with limited insight on consultation approaches. All participants were affiliated with hospitals in one Canadian province; therefore, findings may not be relevant to hospitals in other countries with differing PE practices or health systems.

## 5 CONCLUSION

Through interviews with 40 patient/family advisors, PE managers and clinicians at hospitals in which PE had become pervasive, we identified approaches and strategies that could optimize PE in organizational planning and improvement. Hospitals engaged patients via standing committees, project teams and general and unit/department‐specific PFACs. Hospitals primarily used collaboration (membership on committees/teams/PFACs) or blended approaches, which typically involved consultation to first capture a range of ideas/feedback via surveys or interviews with many patients/family, followed by collaboration to prioritize and elaborate on the most promising ideas/feedback. Participants who employed collaboration emphasized the ability to integrate perspectives in planning/improvement decisions, while those who used blended approaches emphasized the ability to capture many diverse perspectives, and then prioritize and further develop those ideas. Fewer participants used only consultation approaches. Given that patient/family advisors were largely retired Caucasian women deployed to many projects or committees, and issues common to the majority of community members were prioritized, the use of collaboration or blended approaches may not lead to facilities or services that reflect the needs and perspectives of underserved community members. Participants described a wide range of strategies that supported engagement approaches to ensure that diverse perspectives were sought, heard and integrated in planning and improvement decisions. Given little evidence‐based guidance on how best to engage patients in hospital planning and improvement, this research identified concrete strategies that can be implemented in future by hospitals to enhance their PE capacity.

## CONFLICT OF INTEREST

None to declare.

## AUTHORS' CONTRIBUTION

GRB, LM, KS, RU, WW and ARG conceptualized and designed the study. NA and ARG collected and analysed the data. All authors reviewed and interpreted the data. All authors drafted or revised the manuscript, gave final approval of the version to be published and agreed to be accountable for all aspects of the work.

## Supporting information

Supplementary Material

Supplementary Material

## Data Availability

All data are included in the manuscript and supplementary files.
