# Supplementary material for: Approaches to optimize patient and family engagement in hospital planning and improvement: Qualitative interviews
Source: Health Expect. 2021 Mar 24;24(3):967–77. doi: 10.1111/hex.13239 (PMC8235895; doi:10.1111/hex.13239)
Supplement: Supplementary file 1 — Supplementary Material [file HEX-24-967-s002.docx]

**Approaches to optimize patient and family engagement in hospital planning and improvement: Qualitative interviews**

**Supplementary File 1. Interview guide**

| Question | Prompts (selectively applied depending on response to questions) |
| --- | --- |
| Please describe a planning or improvement activity that engaged patients in some way that you were involved in or are aware of | - What was the goal of the activity? - What was the intent of engaging one or more patients? - What units/departments were involved in the activity? - Who led or championed the initiative? - Over what period of time did this activity take place? - What was your specific role? |
| How were patients engaged? | - In what types of decision-making activities (e.g. committees) - Consult – surveys, interviews, focus groups - Co-design – project team or standing committee - When patients were a part of these (surveys, standing committees), what were they actually doing and contributing? - How many patients were involved? - What types of patients were involved? - How did you capture opinions from diverse patients? |
| Why were these patient engagement approaches chosen? | - What are the benefits of those approaches? - Was it based on goal? - Was it based on available resources? - Was it due to prior experience? - Was it due to leader and or coordinator preference? - Are these approaches suitable for patients or professionals? - Are these approaches suitable for hospital planning or improvement? - Do you think some approaches are better than others? |
| What strategies ensured that patient feedback or suggestions were used? | - How was it used or incorporated in decisions? - What specific processes or approaches were used to ensure that patient input was used? |
| What key challenges or barriers did you experience (or arose) while engaging patients and what was done to overcome or solve that problem? | - Identifying/recruiting or preparing patients to be involved? - Preparing clinicians/physicians to be involved? - Establishing roles, responsibilities of those involved? - Related to the way patients were consulted or involved? - Did patient-clinician interaction and communication run smoothly? - Physician involvement or commitment to the PE activity? - Other patient or clinician characteristics? (i.e. were patients hesitant to speak up, were physicians too domineering?) - Lack of organizational resources, such as people or funding |
| Looking back, what would you change or do differently to improve PE? | - Would you involve more patients? - Would you involve more healthcare providers? - What would these individuals do to ensure success in PE? |
