# Supplementary material for: Approaches to optimize patient and family engagement in hospital planning and improvement: Qualitative interviews
Source: Health Expect. 2021 Mar 24;24(3):967–77. doi: 10.1111/hex.13239 (PMC8235895; doi:10.1111/hex.13239)
Supplement: Supplementary file 2 — Supplementary Material [file HEX-24-967-s001.docx]

**Approaches to optimize patient and family engagement in hospital planning and improvement: Qualitative interviews**

**Supplementary File 2. Approaches to PE Data Tables**

**PE was pervasive**

| Theme | PE Managers | Patients | Clinicians | Corporate Executives |
| --- | --- | --- | --- | --- |
| Patients involved in decision-making for all hospital activities | We now have a patient that sits on every care team in the hospital (001 PE manager <100) | - We sit on all committees in the hospital (002 patient/family <100) - Everything that touches a patient pretty much has a patient, a patient council or a patient family member at the table (007 patient/family teaching) - Any kind of initiative at the hospital, they ask for patient representatives to be on it (018 patient/family 100+) - We sit on Board committees; we sit on most, I would say 90% of the hospital committees including department heads (036 patient/family <100) | - It was engrained with our organization that we need to ensure that whatever activity that we partake in that we include the patient family voice (021 clinician 100+) | - We always have a patient or two involved in everything that we do (038 corporate executive teaching) |
| PE allows healthcare workers to see issues through patient lens | - The intent of engaging patients was to ensure that we consider the patient-family experience and that we design the new processes with the patient and family-centred care approach (011 PE manager 100+) | - They [healthcare workers] see things through new eyes (036 patient/family <100) - To have patient-family centred care, you can’t just pay lip service to it, you absolutely have to engage patients (039 patient/family teaching) | - Being able to see our system through their eyes is very informative and leads to better patient experiences which also leads to better outcomes (025 clinician 100+) | **---** |

**Engagement structures**

| Theme | PE Managers | Patients/family members | Clinicians | Corporate Executives |
| --- | --- | --- | --- | --- |
| General and specific PFACs | General PFAC   - We have a Patient Experience Partner Council (011 PE manager 100+) | General PFAC   - I was asked [to be] the Chair of the Patient and Family Advisory Council (005 patient/family teaching) - We’re all volunteer people and I co-chair the Patient and Family Council. That’s a hospital-wide group that looks at very specific things from quality markers to issues within the hospital (007 patient/family teaching) | Specific clinical unit PFAC   - And we partnered with our Parent Advisory Council within NICU (022 clinician 100+) - I lead our Patient and Family Advisory Council within our Mental Health Department (034 clinician 100+) | **---** |
| Standing committees | - They’re [PFA’s] part of the Board and the Board sub-committees (027 PE manager <100) | - I was on the Ethics Committee, they wanted a lot of input from the patients (003 patient/family teaching) - There’s a number of different teams. I go to the Quality Care Committee meetings (014 patient/family teaching) - And we’re [Patient Experience Partners] involved on the quality patient experience committee and a lot of unit councils were on as well (018 patient/family 100+) - I sat on the researcher ethics board (019 patient/family 100+) - PFAC members join different sub-committees, we have someone who’s on the committee for the emergency department, someone who’s on the committee for ethics, finance, etc. (035 patient/family <100) | - They [patients/family members] sit on our Board on our Quality and Safety Committee (008 clinician teaching) - There is a patient on the General Medicine Quality Committee and the Quality Committee…both of those committees had input into what was on the Board agenda (012 clinician teaching) | - We have patient and family advisors embedded in…corporate committees but also at the program level committees (032 corporate executive 100+) |
| Project teams | - We had a small working group that consisted of about 6-people including a patient and family advisor that helped drive and steer the organization in terms of developing the strategic plan (004 PE manager teaching) - So it was more of a task force action group that the PFA was involved in (020 PE manager 100+) | - I was on the steering committee for a way-finding project for the hospital (016 patient/family 100+) - [I was] also involved in the Steering Committee for the hospital’s new e-Health strategy (017 patient/family 100+) - The goal of the stroke council working group was to implement a stroke unit. We had monthly meetings that I was involved in and my role is to bring the patient perspective to these meetings (039 patient/family teaching) | - And the patients were engaged at the steering committee level all the way down to specific working groups in the development of care pathways for these two patient populations (CHF and COPD) (021 clinician 100+) - To identify what an Ontario Health Team collaborative health system would look like… We had a working group committee. There was 30 [patient and family care] partners that were invited to the working group meetings (040 clinician 100+) | Individual patient and family advisors sat on the steering committee that guided through the development of our quality improvement plan (032 corporate executive 100+) |

**Engagement approaches**

| Theme | PE Managers | Patients/family members | Clinicians | Corporate Executives |
| --- | --- | --- | --- | --- |
| Approach selected depended on situation or nature of project | --- | - So co-design is certainly the best but I don’t think that you can use it in every situation (005 patient/family teaching) - I think it depends on the issue. And it depends on how much involvement the people that you’re working with want or are willing or capable based on either time or ability I suppose because there’s logistics that get in the way (007 patient/family teaching) - I think some [approaches] are more appropriate depending on what the task at hand is… I know certain projects are more short term, some are more long term. I think it depends on the nature of the project (015 patient/family teaching) | --- | --- |
| In-person interaction preferred | --- | - They got to see me as a patient and by me telling my story they got to feel what it’s like to go through the hospital system. Where I think if it’s just on paper or they read it it’s a different feel. So to actually have the person in front of you; I think it hits home a little bit more (013 patient/family 100+) - You need to have face-to-face. You need to have certain patients involved in it; in on-going discussions (015 patient/family teaching) - I think that the synergies that you get face-to-face are so much greater than virtual (030 patient/family 100+) - We [PFA’s] would have liked to have a face-to-face meeting with her [PE lead] (036 patient/family <100) | - I think it’s really important regardless of what initiative to do; face-to-face interaction with the patients. You got to develop that trust. You got to develop that intimacy (006 clinician <100) - Certainly being face-to-face is really helpful because there’s a natural back and forth on it (008 clinician teaching) - We wanted to make it as hands-on as we could and our preference is to work with our patient and family advisors in-person (034 clinician 100+) - I think the best was in-person, face-to-face because they [patient partners] gave you great feedback and they felt more comfortable with that (040 clinician 100+) | --- |
| Collaboration rationale | Ensures patient perspectives heard and integrated in decision-making   - And so, that [PFAC revised insight statements] was the way we were able to ensure that the patient voice was front and centre; that it was truly what patients and families were describing or articulating (010 PE manager 100+) - Having people at the table through all of the discussions was extremely important with respect to making sure the voices of patients were there at all times (028 PE manager teaching)   Evidence-based/credible   - So we want to use an evidence-based approach for our project. We wanted to ensure that it wasn’t a one-stop shop engagement opportunity where patients and families had an opportunity to share and then staff go back and do all of the work and interpret and design (010 manager 100+)   Mandated by management   - The Board of Directors setting the expectation that patients and family partnership is important (004 manager teaching) - It’s within our corporate goal. It’s mandatory that they’re partnering with patients and families and they have to be able to demonstrate that, through partnership, they have been successful (010 PE manager 100+) - We made a commitment here at <hospital> that patients are partners. So that approach is used because it’s how the hospital functions (011 manager 100+) | Ensures patient perspectives heard and integrated in decision-making   - What the PFA program does; it adds a very important third leg to the planning and thought that goes into the process of running the hospital and delivering care. The addition of the PFA’s is bringing that patient experience to the table. It’s the third leg of a three legged stool that the PFA provides (016 patient/family 100+)   Evidence-based/credible   - Co-design is actually an established practice…co-design has been shown to be very successful (014 patient/family teaching)   Mandated by management   - Our hospital, well they almost always mandate that patient and family advisors have to be on these kinds of committees (003 patient/family teaching) - Having PFA’s on the REB was an expectation (017 patient/family 100+) | --- | Evidence-based/credible   - We did ask what some of the other hospitals are doing (032 corporate executive 100+) |
| Collaboration examples | - Patients participated in staff introduction videos. The patients helped us to decide how we would introduce ourselves and what would be a value added for them (001 PE manager <100) - So every time we did an analysis where we developed insight statements, those insight statements were presented to the patients and families committee. Then they had the opportunity to revise and then we kept it, it went back and forth, back and forth collaboratively together throughout the process (010 PE manager 100+) | - I sit on a really new innovative program. It was brand new way of delivering services at the hospital, and myself and the other patient partner would for sure say that we influenced the way that that program was developed (014 patient/family teaching) - The coordinator for the REB did a lot of the putting it [consent forms] together but it was based on a lot of the discussion and changes and questions that evolved from the different REB meetings over the time of the last few years (017 patient/family 100+) - Myself and about five or six other patient advisors were involved in the development of an online tool for seniors regarding their health. We met regularly to develop the content that would be included on each page of this site down to the visuals, the sounds of the voice prompts and anything that we felt was relevant (019 patient/family 100+) - One of the projects we’ve worked on was a review of the pre-admission patient handbook. So we took the old version and brought up some of the things that we felt we would have liked to know as a patient or a family member going into the hospital … We went through each thing in the handbook and reviewed whether it was useful to keep there and then they took comments from us as far as other things we would like to see there that maybe weren’t in the handbook to start with (023 patient/family 100+) - There was a palliative care booklet being prepared and we [PFA’s] were asked to do some input into that booklet. We were given a copy to look at and see where we could change things to suit our specific hospital environment. And so we took it away and went over it and it was pretty detailed…we had printed copies so during and between meetings we read it and would find whether there was typos or other things (035 patient/family <100) - When the stroke council working group made a brochure for our stroke unit; I would review it and if there was language for instance that I thought might be complicated for our patients, then I would speak up regarding that. Or if I had questions from the patients perspective as to the meaning of some of the terminology; that future patients might find confusing, then certainly I would bring that up… we had monthly meetings and my role as a patient-family advisor by definition is to bring the patient perspective to these meetings (039 patient/family teaching). | - The patient advisors were part of the working group, we would meet once a month and develop the standardized care pathway for the program. If something didn’t work we would actually tweak the process and they were really involved in that. And then with the evaluation of the program, we would meet again on a bi-monthly basis and review any performance metrics and they again, were involved (021 clinician 100+) - So in the NICU we took an opportunity to revamp a parent information booklet. We actually had parents help us define what the key elements of the information brochure and/or platforms would be in terms of paper-based and electronic version. They helped us to develop the table of contents and what the important pieces were that needed to be included (022 clinician 100+) - We were looking at the creation of a new care pathway. So we did an experienced-based design work with patients and families to help inform us and really try and work with patients and families around how they would look at their pathway going forward and learn from their experiences. We had a governance structure around it and for example, a steering committee. And we would have patient experience partners that are part of those committees, helping to inform our processes going forward. So they would participate in committee meetings and be active participants as we made decisions and talked about various things (025 clinician 100+) - So we thought that what would be the most helpful is to bring all our thoughts to the table and then brainstorm as a group with PFA’s to decide what would be best and most appropriate and most helpful in our waiting rooms psycho-education material (034 clinician 100+) | --- |
| Consultation rationale | Reaches many patients   - We attended five or six summer festivals and choosing that style allows us to get many perspectives instead of just one or two. We had thousands and thousands of points of data (004 PE manager teaching) - We sent a survey to that group [virtual patient focus group] as well as our entire community of patient partners which is about 130 permanently that are active. We really wanted to get broad engagement… it needed to be reflective of different voices and experiences and potentially those that are not traditionally sought out (028 manager <100)   Captures diverse perspectives   - A few [patient/family advisors] at the table is a heavy burden for those individuals to carry the voice of all patients (028 PE manager teaching)   Efficient way to rapidly gather information in support of decision-making   - We wanted to know with the stuff we’re putting in that day, did it make sense? And if it didn’t what do we need to change to improve it? So the intent was to get actionable information that we could tweak each day (020 PE manager 100+) | Reaches many patients   - The broader the input, the better it is…it’s getting as much information from as many people as possible (030 patient/family 100+)   Efficient way to rapidly gather information in support of decision-making   - They [staff] want to get information from patients faster so that they can look at making decisions about improvements more immediately (018 patient/family 100+) - The phone surveys is just quick and easy (026 patient/family 100+) | Reaches many patients   - We were trying to reach out to more people (040 clinician 100+) | --- |
| Consultation examples | - Our hospital developed a patient declaration of value … And what we did is we had world café style where patients and families were able to talk about what was important to them when receiving care (010 PE manager 100+) - We’ve done more focus groups; going out of the hospital to get patient feedback. And I’ve done focus groups with small groups through a specific topic with people that I’ve met through the patient relations file that don’t become advisors (011 PE manager 100+) - A group of about 200 patients and families who have had care in the organization have agreed to be a part of a virtual group where they would receive…a few surveys a month on various topics (028 PE manager teaching) | - So we [PFA’s] got lots of emails asking if the information that they were putting out [about the healthy food initiative] was good. We got consulted about the policies that were put in place to manage this whole thing… They asked for our opinions on some questions… to see what we thought of it (003 patient/family teaching) - They [the hospital] had several booths set up for two weeks in the main corridor. They invited patients or whoever was in the main lobby. So getting their feedback to enable creating this new strategic plan (005 patient/family teaching) - It [virtual survey] was across the entire system; they have a virtual group that are all patients and family members that they tap into when they want to do a survey of a process or an idea or a concern and then that information comes back (015 patient/family teaching) - We were given a certain number of questions and we were calling people just to get feedback. They [patients] would give their feedback on the phone. We [Patient Experience Partners] would enter that information and then that would go to the coordinators of the project and it would also go the managers of the unit. And that was all about trying to improve the quality of care (018 patient/family 100+) - We would call patients, usually we try to call them within 2-days of their discharge to ask them the questions. They were general questions that the hospital wanted to get a leg up on to see if we could improve where the public was saying that we had areas that we could improve upon (026 patient/family 100+) | - Surveying friends, family, staff, patients, and people on the PFAC committee. We had originally 32 suggestions for patient and family-centred care, twiddled it down to 17 and out of the 17, enhancements to hospital staff identification badges is one of them (006 clinician <100) - We pulled together a program to refresh our seniors care framework within the organization. Surveys were sent out to both internal and external stakeholders which included patients and families. So we started with surveys, then we actually did guided interviews with specific stakeholders and again, patients and family advisors were included in that (021 clinician 100+) - I directly manage a post discharge contact program for high risk patients. So we have an electronic survey that guides the conversation and we record the details into the electronic survey,…we ask them for feedback around their stay … we share all their feedback with the individual frontline teams (031 clinician 100+) | --- |
| Blended approaches rationale | Consultation gathers a wide range of perspectives, then co-design provides deeper insight on those ideas and which might be prioritized because they would lead to the biggest improvements   - So you can’t collaborate with the whole community but you can get their input and then bring it into an environment where collaboration is possible (004 PE manager teaching) | Consultation gathers a wide range of perspectives, then co-design provides deeper insight on those ideas and which might be prioritized because they would lead to the biggest improvements   - I think we [Patient Partners] could bring a certain perspective and the virtual survey group could bring another… we [Patient Partners] would break into groups and review the suggestions from the survey group to make sure that it was captured (015 patient/family teaching) - At this time it’s sort of gathering all this information and I guess as they move forward and they have a few patient and family advisors that will work on it; I think they will get more in depth on the project (030 patient/family 100+) | --- | --- |
| Blended approaches examples | - We involved patients and the families in what they wanted to put on whiteboards and what they would look like. We did informal surveys with patients in the hospital. We brought different samples of the whiteboards to our PFAC for them to have input (001 PE manager <100) - The goal was to re-design the model of care in the Emergency Department… So we’ve had patients involved in that plus our Patient Experience Council advises on different aspects of it… We did rounding, so the Chief Nursing Executive was asking people questions about their experience in real-time; given the changes that are happening. So she’s also looking for feedback from people who are receiving care as well… and then there’s meetings that have different voices around the table. So those groups advised on different aspects of it (011 PE manager 100+) - The overall results came back to the patient and family experience steering committee to inform whether we needed to make some tweaks to the process that we would then trial in our next PDFA cycle (024 PE manager 100+) | - There was a draft of the [Patient Declaration of Values] document we pulled together and the committee [of PE staff and PFAC members] broke into teams and we went across the sites. We went to clinics. We went to medical offices. We went to reception areas. We sat down and talked to patients as they were getting chemotherapy and we shared what we were doing. Was there anything that resonated with them? Was there anything that we had missed? We took it all (015 patient/family teaching) | - We engaged the PFAC in the information they would want to see [on the communication whiteboards] and then we did a lot of engagement about how that information would be organized and how the whiteboards should look so that they’re user-friendly for both patients and for their families… And then we had some boards created and put them up in one of the wards over a period of time and asked for feedback… We had data collection forms for what patients and their families thought of when we prototyped the patient communication whiteboards on the ward (012 clinician teaching) - So one of the goals is to work collaboratively with our patients and families and care partners within the hospital to identify what an Ontario Health Team collaborative health system would look like… We had a working group committee. There was 30 patient and family care partners that were invited to the working group meetings. So they would physically come to the meetings and help us identify specific questions… We also had a small survey developed and we reached out to further community partners and community members to ask them of the questions on the survey. So they, they were our consultants (040 clinician 100+) | - We have a post contact discharge program. We have nurses that contact the patients following discharge; They ask some patient experience related questions that we leverage when we’re building our quality improvement plan… We outlined the key themes or priorities from a patient safety perspective. Then we outlined and layered that with what we heard from our patient relations data surveys. And then on top of that, we outlined what the PFA’s have heard through their various committees and then also talking to patient and family members. And then we [steering committee with 2 PFA’s] actually identified common themes and did a prioritization exercise where we scored which ones would be in highest priority (032 corporate executive 100+) |

**Strategies to optimize engagement**

| Theme | PE Managers | Patients/family members | Clinicians | Corporate Executives |
| --- | --- | --- | --- | --- |
| Engage diverse patients | Aim for diversity in characteristics   - We believe in making sure that the most marginalized individuals have represented voices at the table…one of the things in terms of how we’ve been successful with being able to recruit these types of individuals is that we have a strategy around recruiting for diversity. So we specifically are looking for folks that represent the health disparities in our community and engage them (010 PE manager 100+) - We’re basically recruiting people for committees and projects that are approved through the Board and part of our quality improvement plans and then we make sure that we have patient-family voice and the experience data also feeds into that (011 PE manager 100+)   Employ various recruitment strategies to achieve diversity   - We did a call-out to our PFA’s to see who might be interested in it and then we took the two that basically responded first (020 PE manager 100+)   Patient/family advisors were largely retired persons   - It tends to be the retired community that comes forward to be part of the patient and family advisory committee (027 PE manager <100) | Aim for diversity in characteristics   - They tried to diversify the group [PFAC]. I think it really does make a difference (035 patient/family <100)   Employ various recruitment strategies to achieve diversity   - The way our process works is a call goes out to the group [PFAC] to ask if you’re interested in the committee. We respond with a little thing about why we might want to be on that committee and what our expertise might be. And then I believe those little things go to the department manager or whoever is asking for representation and they decide (003 patient/family teaching) - They’ll put out all the call-outs for anyone who’s interested in participating in a particular project. And then volunteers will respond to them and then get placed on projects (007 patient/family teaching) - Sometimes there was just a hey, we need a couple of people to help us work on such and such, please let us know if you’re interested … You know they were very kind in how they asked. It was if you want to. If this suits you, let us know. So stuff was optional (014 patient/family teaching) - What would happen is I get an email that says, here is a PFA opportunity; people are looking for you know help with “x”, whatever “x” is (016 patient/family 100+) - We have had discussions, we put in adverts in the paper, on the news, on Facebook (035 patient/family <100) - A lot of our recruiting often times is by word of mouth and we’ve tried newspaper ads, the last one we got through Facebook…you know it’s those kinds of things; how do you reach the biggest population? (036 patient/family <100) - What they’ll do is they’ll put out the requests for patient-family advisors via email. So you get a request that says, they’re looking for a PFA (039 patient/family teaching)   Patient/family advisors were largely retired persons   - When you get to a certain age and when you’re retired; the last thing I want to do is to sit down and read books all the time. That’s not what I need and the hospital involvement allows me to feel like I’m doing something to make a difference for other people in a small way (019 patient/family 100+) - Because I’m retired I’m able to give the time to things (023 patient/family 100+) - Our team has different backgrounds. Okay, so some are retired nurses. Some are retired teachers and principals (036 patient/family <100) | Aim for diversity in characteristics   - What we did was we looked for volunteers from our patients as well as the family members and was very lucky to engage two family members; one for COPD, one for CHF of patients with both of these chronic progressive diseases. So they really had a breath of lived experience that they could share (021 clinician 100+) | Employ various recruitment strategies to achieve diversity   - So we struck a working group and we have a project charter and then what we do in our hospital is we reach out to our patient and family advisory council. We sort of put together a little job description of what we’re looking for in terms of patient or family involvement in the working group. And they put that out to all of our PFA’s and then interested people apply to participate in the working group (038 corporate executive teaching)   Patient/family advisors were largely retired persons   - I would say for the majority of our patient volunteers they are retired (038 corporate executive teaching) |
| Prioritize what benefits many | Blended approach of consultation then co-design   - We were really trying to be driven by the data from our survey and post-discharge phone calls, and then validating that with the experiences of our patient and family partners to dig a little bit deeper on some things that would have the biggest impact on improving our results (024 PE manager 100+) | Chose projects that benefit the majority   - A lot of suggestions come to PFAC, but if they are more individualized, we try to triage that because it’s not about one, it’s about everybody. We try to talk about who is our catchment and who are we benefiting (002 patient/family <100)   Used perspectives expressed by the majority   - We had reports come back from residents…we knew that it wasn’t biased on a few people, it was spread out over a great number of people (002 patient/family <100) | Used perspectives expressed by the majority   - And then when the structured reviews came back from patients and their families we had to set a priority, we’re going to take everything that is said by more than you know “x’ percentage of patients and we’re going to use that (012 clinician teaching) | --- |
| Match patients to projects | Deploy those with PE experience/skill   - We also create opportunities for the patient partners to be involved in quick development, and some of them can really show some promise in that corporate approach and those are the folks that we often recruit into the steering committee (024 PE manager 100+) | Deploy those with PE experience/skill   - Sometimes we were selected because of other projects we had worked on. I mean they [PE managers/staff] have a good sense of our skills at this point. There’s a large number of patient partners but there seems to be a group that does a lot of different kinds of projects and so they know who’s got good analytical skills and good communication skills. So we were sought. We were recruited specifically (014 patient/family teaching) - I think these were cultivated requests within the hospital. I think it was targeted specifically with people who would have some familiarity and comfort with the activity (029 patient/family 100+) - There’s a number of factors taken into consideration [when recruiting PFA’s for a project]; such as which committees you’ve been on in the past (039 patient/family teaching)   Match patient/ family experience or characteristics to PE project   - There was a request for patient advisors [to design aging-well tool for seniors]. We were given an outline and there was about 5 on the committee that was formed, and we were all seniors. So we certainly were thinking from experience (019 patient/family 100+) - And because I had personal experience; don’t send me to the NICU, I have no idea about babies and I’ve never been there, right? So I can’t give you any insights into that at all because I have no experience there, right? So the fact that I had just recently had a personal connection to the unit (033 patient/family 100+) - And basically what they ask for generally is people who have had a background as a patient in those areas. So for instance, when things come out in the neuro area or the cancer area I wouldn’t apply because my background there as a patient just doesn’t exist (039 patient/family teaching) | Match patient/ family experience or characteristics to PE project   - So for us we’ve been very lucky because we’ve had parents who have graduated NICU here with us. So they’re removed from the care environment and it provides them then with an opportunity to reflect on what it was like and in a very objective and like non-confrontational or non-threatening way to be able to provide insight for us that they may not have been able to or felt safe enough to provide in the moment; they have the actual experience (022 clinician 100+) | --- |
| Train participants | --- | Train patient/family for role of advisors   - We usually have an education session for about a half an hour before we get into the meeting (036 patient/family <100) - They trained us, they used us, they educated us and then when they really needed us, here we are (014 patient/family teaching) - We’re [patient partners] offered all the education that’s been offered to staff (018 patient/family 100+)   Train healthcare workers on how to collaborate with patient/family advisors   - The staff and leaders received training on how to effectively engage with patient partners (029 patient/family 100+) | Train patient/family for role of advisors   - They are trained during orientation and then have, with the interest in the program, a full day of training and then continued engagement throughout the program (037 clinician <100) | Train patient/family for role of advisors   - We have our PFCC department that already trains and vets and looks after patient and family advisors. So they all register, they go through training. They are sort of vetted in terms of understanding the kind of work that we’re trying to accomplish with them (038 corporate executive teaching) |
| Ensure patient perspectives inform decisions | Include a critical volume of patient/family advisors   - About four years ago we established our first patient and family experience steering committee. The initial membership, the staff greatly out-numbered the number of patient family partners. Over the last few years we’ve decreased the number of staff on the committee, increased the number of patient family partners on the committee (024 PE manager 100+)   PFAC review of standing committee or project team work   - We had striked a steering committee. Their initial role was do an environmental scan and pull together all of the publicly stated declarations to do a review … The next step was create insight statements;… Those insight statements were brought to the PFAC for further iteration (010 PE manager 100+)   Philosophical commitment to respect/value patient perspective   - We’ve evolved and developed an understanding that any member around the table would have equal input or equal weighting to their opinions. So whether or not it was a frontline staff at the table or a patient and family advisor or the vice-president or a physician (004 PE manager teaching) - I feel that their [patients] viewed as equal influencers in terms of the planning (11 PE manager 100+) - We’ve come to value that. They are like team members. It’s built into the organization (020 PE manager 100+) | Quorum requires at least one patient/family advisor   - There’s usually two of us [patient/family advisors on standing committees]. One of the requirements of the REB is that to have a proper quorum you need to have one patient/family advisor at the meeting (017 patient/family 100+)   PFAC review of standing committee or project team work   - The healthy eating committee came to the patient-family advisory committee as a whole twice and educated the whole group on what was happening and asked for their opinions (003 patient/family teaching) - I would bring information back to our PFAC Council, as new information we were allowed to share; so we had monthly meetings. I would bring the group up-to-date, etc., (005 patient/family teaching) - I would report back to the Patient and Family Council about what is going on in the General Medicine Quality Committee…and sometimes I’d be seeking out advice (007 patient/family teaching) - So there was a small working group that developed the questions. And then it went through the patient and family experience committee … they would run it by us (026 patient/family 100+)   Patient/family advisor feedback loop   - Somebody comes back to you and says, here’s how your comments changed what we did. It’s a very simple feedback loop but it’s made a big difference (014 patient/family teaching) - We would have a meeting one week and the next week they would come back with an document. And then we would break into groups and review the suggestions to make sure that it [patient input] was captured. Then we would have a more fulsome discussion. I think they’ve retained that information and I think they utilized that information in every way possible to ensure that patient’s voice was embedded in this document (015 patient/family teaching) - Often at the end of the review of a project we would go back to the project leader saying, here are the things that we asked you to look at or consider before continuing. And that’s where we would actually itemize any changes raised by anybody; not just the PFA but anybody on the Board. So yes there is a closed loop on that (017 patient/family 100+) - The researcher would bring back drafts and we would review the different topics as we were going through them. She would bring back the topics and the input that we had and we may adjust them. It was particularly thorough (019 patient/family 100+) - When we [patient/family advisors] made those suggestions, they were taken away and then at the next meeting they would hand the draft out and we’d go over it to see which of our suggestions had been included (035 patient/family <100)   Philosophical commitment to respect/value patient perspective   - The respect for patient/family advisors within the whole organization is very conducive to them [healthcare workers] listening to us and taking our advice (003 patient/family teaching) - We were actively asked in the [PFAC] meetings for opinions from the perspective of the patient and that is our rule. When we’re onboarded as PFA’s it’s made very clear to us that our role is to respect and reflect the voice of the patient (016 patient/family 100+) - Everybody had an equal say at that table and their comments were very well received so nobody was hesitant to speak up (023 patient/family 100+) | PFAC review of standing committee or project team work   - A select few patient and family volunteer individuals [worked on a committee organizing the elder life program] but it was brought to the broader PFAC for comment on policies/procedures, etc., (037 clinician <100)   Patient/family advisor feedback loop   - We put all of the information together in a patient experience map. So the nurse did do some follow up with the patients to show them the map and really verified that this was the things that they were saying (025 clinician 100+) - We’re sending that out so that they [patient/family advisors] look at it and make sure that it reflects what they’ve said (040 clinician 100+)   Philosophical commitment to respect/value patient perspective   - We come to the table with the philosophy that our family advisors are the experts (008 clinician teaching) - They [parent advisors] were equal partners at the table (022 clinician 100+) | PFAC review of standing committee or project team work   - So we have two PFA’s representing the steering committee but the PFA’s would gather information from other PFA’s embedded in other committees as needed… So they were reaching out to the remainder of the patient and family advisors (032 corporate executive 100+)   Philosophical commitment to respect/value patient perspective   - Everybody that’s engaged we’re appreciating them and valuing their time and commitment (032 corporate executive 100+) - So they’re [patients] equal partners in that work (038 corporate executive teaching) |
| PE manager/staff champions | Skilled PE managers/staff   - They have really key skills around hearing, deep listening and reflective listening skills, and know the importance of being able to hear and integrate (028 PE manager teaching) | Skilled PE managers/staff   - We have a very good leader in the management position for PFA’s who is very good and they always take our suggestion (003 patient/family teaching) - They [PE managers] ensured that all the information was compiled; that all the suggestions that we [PFA’s] had at the table and then the suggestions that came from the larger patient survey group; all of those ideas were captured (015 patient/family teaching)   Proactive standing committee/project team Chairs   - Some of the people facilitating the group were very pointed that at every stage; there were continual check-ins. These were long meetings; 3-4 hours but every half hour I know I would be asked if that makes sense. Did I have any questions (29 patient/family 100+) | --- | Proactive standing committee/project team Chairs   - The chair of the working group made sure that all members are actively participating and that their voice is heard (038 corporate executive teaching) |
| Link with Board of Directors | Board member on PFAC   - Having a Board member sit on the PFAC, and bring those minutes to the Board [Quality] Committee and to the full Board ensures that if they need different equipment or whatever, that it’s not just being minuted in a meeting and then never done (001 PE manager <100) | Patients on Board/Board Committees   - We [patient/family advisors] also have voting rights on the Board committees (036 patient/family <100) | Accountable to Board   - Being accountable to the Board…to report that there has been implementation and change (037 clinician <100) | --- |

**Engagement challenges and recommended solutions**

| Theme | PE Managers | Patients/family members | Clinicians | Corporate Executives |
| --- | --- | --- | --- | --- |
| PE challenges | --- | Diversified patient representation   - As we’re getting into challenges for a PFA program; I think that basically the biggest challenge is how do you make sure that you’ve got the optimal representation coming from a PFA standpoint (016 patient/family 100+) - I’m a semi-retired white male and I don’t really feel I represent my community at the hospital. There needs to be more effort made to bring people to the table that aren’t necessarily system savvy and engagement literate (029 patient/family 100+) | Diversified patient representation   - We often get the same experience [from PFA’s]. So it’s difficult when we’re trying to plan something for a marginalized population for example, to make sure that we’re engaging with patients with that experience. If you’re a white retired teacher with breast cancer, we’ve got lots of that at the table (038 corporate executive teaching) | --- |
| PE recommended solutions | Engage patients earlier in activities via co-design   - So the initial conversations with the charge nurses and managers did not have patient and family partners at the table. And that was a choice and I think going forward I’d love to be in a different position where for those initial conversations we can have patient and family partners at the table (024 PE manager 100+) | Recruit larger pool of patients   - We’ve had to increase the number of advisors because we’re being asked to be involved in many different projects and we just don’t have enough people (005 patient/family teaching) - I would have liked if the situation had allowed there to be more than one patient advisor at the table (007 patient/family teaching)   Engage patients earlier in activities via co-design   - The people in this particular clinic have decided they’re going to do “x” and “y”. I thought the new decisions were not great. I wish there was more opportunity for co-design because that could have saved some problems that we were trying to solve later (014 patient/family teaching) - So one of the things we push hard on is encouraging the hospital researchers to identify a PFA right at the beginning…before they even submit a study to understand the patients perspective as they go through building their study (017 patient/family 100+) | Recruit larger pool of patients   - Consider gathering a larger group of patient and family advisors so we have a bigger pool of resources (034 clinician 100+) - I would continue to involve more [PFA’s or patient volunteers] … And on an on-going basis (037 clinician <100) | Engage patients earlier in activities via co-design   - We’ll have already done all the work and we’ll bring it to a PFAC group for review and we could have saved so much time if they’d been involved at the very beginning (038 corporate executive teaching) |
